# An Industry-Scale Mass Marking Technique for Tracing Farmed Fish Escapees

**DOI:** 10.1371/journal.pone.0118594

**Published:** 2015-03-04

**Authors:** Fletcher Warren-Myers, Tim Dempster, Per Gunnar Fjelldal, Tom Hansen, Stephen E. Swearer

**Affiliations:** 1 School of BioSciences, University of Melbourne, Parkville, Victoria, Australia; 2 Institute of Marine Research, Matre Aquaculture Research Station, Matredal, Norway; Scottish Association for Marine Science, UNITED KINGDOM

## Abstract

Farmed fish escape and enter the environment with subsequent effects on wild populations. Reducing escapes requires the ability to trace individuals back to the point of escape, so that escape causes can be identified and technical standards improved. Here, we tested if stable isotope otolith fingerprint marks delivered during routine vaccination could be an accurate, feasible and cost effective marking method, suitable for industrial-scale application. We tested seven stable isotopes, ^134^Ba, ^135^Ba, ^136^Ba, ^137^Ba, ^86^Sr, ^87^Sr and ^26^Mg, on farmed Atlantic salmon reared in freshwater, in experimental conditions designed to reflect commercial practice. Marking was 100% successful with individual Ba isotopes at concentrations as low as 0.001 µg. g^-1^ fish and for Sr isotopes at 1 µg. g^-1^ fish. Our results suggest that 63 unique fingerprint marks can be made at low cost using Ba (0.0002 – 0.02 $US per mark) and Sr (0.46 – 0.82 $US per mark) isotopes. Stable isotope fingerprinting during vaccination is feasible for commercial application if applied at a company level within the world’s largest salmon producing nations. Introducing a mass marking scheme would enable tracing of escapees back to point of origin, which could drive greater compliance, better farm design and improved management practices to reduce escapes.

## Introduction

Farmed fish escapees from sea-cage aquaculture are perceived as a serious threat to wild fish populations as they can cause damaging ecological impacts. These include transfer of diseases to wild fish [1], introduction and establishment of escapees as exotic species [2], competition between escapees and wild stocks [3,4], and outbreeding depression through genetic mixing of wild and farmed populations from hybrid crosses [5,6].

Atlantic salmon (*Salmo salar*) is the most commonly occurring farmed fish escapee from sea-cage aquaculture [7]. For instance, 4.6 million salmon were reported to have escaped from Norwegian fish farms from 2001–2012 (http://www.fiskeridir.no/) and escapes occur in all salmon farming countries. Although most farmed escaped salmon disappear, never to be observed again [8–10], some survive and migrate into rivers and onto the spawning grounds of native populations [11]. As a result of farmed salmon successfully spawning with wild salmon, genetic changes have been observed in native salmon populations in Ireland [12] and Norway [13], with introgression of farmed salmon estimated at 0–47% for 20 native populations spanning the entire Norwegian coastline [14]. Introgression of farmed salmon in native populations is of significant concern because their offspring display reduced survival in the wild compared to wild salmon [3,15,16], and may also disrupt local adaptations [17].

Although fish farmers in many jurisdictions are obliged to report escapes, in some cases, escapes of farmed fish are not reported to the authorities. Under reporting is problematic, as without an understanding of why fish escape through technical investigations of escape causes, improvements to technical standards cannot be made rapidly [7]. Detecting escapees and determining the farm they originated from is possible through DNA-based methods [18] or fatty acid profiling [19], although these methods do not identify the farm in all cases. As an alternative, a permanent coded mark or tag for all farm fish applied at an industry scale would allow for a fail-safe method to trace escapees to their point of origin. Numerous methods currently exist to mark fish (e.g. adipose fin clipping and physical tags [20]; otolith thermal marking [21]; fluorescent markers [22]), but all fail in one or more aspects related to the ability to deliver 100% traceability to point of origin, fish welfare considerations or cost-effectiveness at industry scale.

Here, we advance a recently developed marking technique for identifying and tracing farmed Atlantic salmon escapees using stable isotope otolith fingerprint markers, delivered during vaccination [23], by testing multiple combinations of seven enriched isotopes over a concentration gradient, to determine if the technique can be feasibly applied at full industrial scale where up to 500 million fish require marking each year. Marking during routine vaccination could effectively and accurately mark all farmed fish in commercial facilities with no additional manual handling or labour costs. Typically, Atlantic salmon are routinely vaccinated during the parr stage with multi-vaccines against a range of pathogens [24]. Otolith fingerprinting during vaccination is 100% successful using enriched stable isotopes ^137^Ba and ^86^Sr at high concentrations, and marginally successful (0 to 35%) with enriched ^26^Mg [23]. Otolith fingerprinting via larval immersion on other species suggests that the use of additional stable isotopes of Ba, Sr and Mg could produce over 100 possible otolith fingerprint combinations via vaccination [25,26]. Whether these combinations produce viable marks and what minimum dosages are possible for cost-effectiveness for marking during vaccination must be determined to make this marking technique financially feasible for industry-scale application.

Here, we tested seven enriched stable isotopes (^134^Ba, ^135^Ba, ^136^Ba, ^137^Ba, ^86^Sr, ^87^Sr, and ^26^Mg) at 4 concentration levels (1, 0.1, 0.01 and 0.001 μg. g^-1^ fish) in fingerprint combinations of 1, 4 or 7, which could provide 127 unique marks. To make the experiment industry-relevant, we followed standard commercial farming practices for salmon. Moreover, we monitored the health and welfare of all marked fish until they grew to harvest size (5 kg) to determine if marked fish had similar condition and welfare to unmarked control fish and that the concentrations of stable isotopes of Ba, Sr and Mg used in this study are harmless for farmed salmon.

## Methods

### Otolith fingerprint marking with enriched stable isotopes during vaccination

**Ethics statement.** This study was conducted in accordance with the laws and regulations of the Norwegian Regulation on Animal Experimentation 1996. The protocol was approved by the Norwegian Animal Research Authority (Ethics permit number: 6176).

**Experimental location and fish.** The experiment was conducted at the Institute of Marine Research, Matre Research Station, in Masfjorden, western Norway (60°N). A total of 650 Atlantic salmon (AquaGen strain) parr (mean ± SE: fork length = 19.8 ± 0.04 cm; weight = 103 ± 0.6 g) were used in the experiment. All fish were pit tagged with 11 mm Trovan ID 101 tags (BTS Scandinavia AB, Sweden) four months prior to the experiment, and reared in freshwater tanks buffered with saltwater to a salinity of 0.7 g NaCl.L^-1^ in standard commercial hatchery conditions. Fish in all treatments were of similar length and weight at day 1 of the experiment (one-way ANOVA; length; F_12, 649_ = 1.32, *p* = 0.2, weight; F_12, 649_ = 0.87, *p* = 0.6).

**Experimental design.** We tested three combinations of the enriched stable isotopes, ^134^Ba, ^135^Ba, ^136^Ba, ^137^Ba, ^86^Sr, ^87^Sr and ^26^Mg (Oak Ridge National Laboratory; www.ornl.gov) at four concentrations (1, 0.1, 0.01, or 0.001 μg of each isotope per g of parr average weight) to determine the minimum isotope concentrations required to ensure 100% mark success of isotope fingerprint tags delivered during vaccination [23]. Atlantic salmon parr (50 per treatment) were injected with the multi vaccine MINOVA 6 (NORVAX MINOVA 6, Global Aquatic Animal Health, Bergen, Norway) that contained either: 1) no isotope enrichment; 2) enriched ^137^Ba; 3) a combination of enriched ^135^Ba, ^136^Ba, ^137^Ba and ^86^Sr; or 4) a combination of enriched ^134^Ba, ^135^Ba, ^136^Ba, ^137^Ba, ^86^Sr, ^87^Sr and ^26^Mg (Table 1).

**Table 1:** Experimental design.

Factors		Injection	Sample sizes		
Isotope fingerprint mark	Concentration (μg. g^-1^ fish)	Total isotope used (μg) in 0.1 ml injection per 40 g fish	Total treatment (N)	Growth analysis (N)	Otolith analysis (N)
No fingerprint (control)	0	0	50	50	10
^137^Ba	1	40	50	50	10
^137^Ba	0.1	4	50	50	10
^137^Ba	0.01	0.4	50	49	10
^137^Ba	0.001	0.04	50	50	10
^137^Ba, ^136^Ba, ^135^Ba, ^86^Sr	1	160	50	50	10
^137^Ba, ^136^Ba, ^135^Ba, ^86^Sr	0.1	16	50	50	9
^137^Ba, ^136^Ba, ^135^Ba, ^86^Sr	0.01	1.6	50	50	10
^137^Ba, ^136^Ba, ^135^Ba, ^86^Sr	0.001	0.16	50	50	10
^137^Ba, ^136^Ba, ^135^Ba, ^134^Ba, ^87^Sr, ^86^Sr, ^26^Mg	1	280	50	50	10
^137^Ba, ^136^Ba, ^135^Ba, ^134^Ba, ^87^Sr, ^86^Sr, ^26^Mg	0.1	28	50	49	9
^137^Ba, ^136^Ba, ^135^Ba, ^134^Ba, ^87^Sr, ^86^Sr, ^26^Mg	0.01	2.8	50	50	10
^137^Ba, ^136^Ba, ^135^Ba, ^134^Ba, ^87^Sr, ^86^Sr, ^26^Mg	0.001	0.28	50	49	10

Design of the experiment to test mark success and strength through introducing isotope fingerprint combinations of one, four or seven enriched stable isotopes at four concentrations during routine vaccination. Sample sizes of fish per treatment and those used for growth analyses and otoliths analyses are shown.

Enriched stable isotopes in powder chloride form (BaCl_2_, SrCl_2_ & MgCl_2_) used for the isotope enrichment treatments were first dissolved in Milli-Q water to make standard stock solutions of each isotope combination (i.e., 1, 4, or 7 isotope markers). The required isotope combination-by-concentration solutions were then mixed firstly by pipetting the appropriate amounts from the standard stock solutions into a 1 ml eppendorf tube and then mixing this solution with the MINOVA 6 vaccine on the day of vaccination. Final solutions were agitated for 30 seconds using a Virvel Mixer (Heidolph Instruments Gmbh & Co.KG) to ensure a stable solution. Injections (0.1 ml per fish) were given into the abdominal cavity, approximately 20 mm behind the pectoral fin on the ventral side of parr using a standard commercial vaccination gun (Socorex Swiss-167; www.socorex.com) fitted with a 5 mm, 27 gauge vaccination needle.

On the day of vaccination, fish were anaesthetised with Benzoak VET (dose 0.2 ml L^-1^ of clean hatchery water), identified by their PIT tag number, then weighed, measured (fork length) and injected. After injection, fish were placed into one of five 1000 litre tanks with equal interspersion of individuals among treatments within each tank (i.e. 130 fish per tank, 10 from each treatment). The fish were reared under a 12 h light: 12 h dark photoperiod for the first six weeks post injection before being switched to 24 hours continuous light to induce smoltification. To monitor differences in growth and condition among treatments, 90 days post-injection, all fish (n = 50 per treatment) were anaesthetised, weighed, and measured (fork length). At this time, a randomly selected sub-sample of 10 fish per treatment, were euthanized by anaesthetic overdose and their otoliths were removed for isotope analysis. Sagittal otoliths (mean ± SE: maximum diameter = 3.3 ± 0.1 mm) were cleaned of any adhering tissue, air dried, and stored individually in plastic tubes. Remaining fish were transferred to a sea cage farm and grown to commercial harvest size (~ 5 kg, 570 days post-injection), then humanely culled with a quick blow to the head, measured (fork length) and weighed, to assess condition at harvest.

**Otolith preparation.** Otoliths were prepared as per Warren-Myers et al. [23]. Sagittal otoliths were cleaned of any remaining organic tissue by immersing in a solution of ultrapure 15% H_2_O_2_ buffered with 0.1 M NaOH. Following immersion, otoliths were ultra-sonicated (Sonic Clean 250HT) for 5 minutes and then left for 6 hours in the cleaning solution. The cleaning solution was then aspirated off and the otoliths were transferred through three Milli-Q water rinses, each of which consisted of 5 minutes of ultra-sonification and 30 minutes resting time. Otoliths were then air dried in a laminar flow bench for at least 24 hours. Once dry, one otolith per fish was fixed, sulcus side down, onto gridded microscope slides using quick dry cyanoacrylate glue.

**Otolith analysis.** Stable isotope analyses were done on a Varian 7700x Inductively Coupled Plasma Mass Spectrometer (ICP-MS) fitted with a HelEx (Laurin Technic and the Australian National University) laser ablation (LA) system constructed around a Compex 110 (Lambda Physik) excimer laser operating at 193 nm. 612 and 610 NIST (National Institute of Standards and Technology) glass standards doped with trace elements at known concentrations was used to calibrate the system. External precision estimates (%RSD) based on 20 analyses of a MACS3 microanalytical carbonate standard were as follows: ^134^Ba:^138^Ba = 7.37; ^135^Ba:^138^Ba = 0.81, ^136^Ba:^138^Ba = 4.51, ^137^Ba:^138^Ba = 0.72, ^86^Sr:^88^Sr = 0.94, ^87^Sr:^88^Sr = 1.16 and ^26^Mg:^24^Mg = 0.60. Otoliths were run in blocks of 16 samples selected randomly from all treatments and bracketed by analyses of the standards. Samples and standards were analysed by vertically profiling in time-resolved mode, using a stationary laser with a spot size of 157 μm, an energy setting of ~ 60 mJ and a repetition rate of 10 Hz. Ablation was performed under pure He (200 ml/min) to minimise re-deposition of ablated material and the sample was then entrained into the Ar (0.95 ml/min) carrier gas flow to the ICP-MS. Using this method, we were able to quantify the concentrations of 134Ba, 135Ba, 136Ba, 137Ba, 138Ba, 86Sr, 87Sr, 88Sr, 24Mg, 26Mg and ^43^Ca in the outer region of salmon pre-smolt otoliths. Data were processed off-line using a specialised MS Excel template which involved a low pass filter to remove any spikes (a single acquisition value >2x the median of the adjacent acquisitions), smoothing (a running average of 3 acquisitions) and blank subtracting functions (an acquisition = a single measure of an isotope ratio while vertically profiling in time-resolved acquisition mode). A correction factor (*K* = *R*_*true*_ */R*_*obs*_, where *R*_*true*_ is the naturally occurring isotope ratio and *R*_*obs*_ is the average isotope ratio measured in the NIST 612 and 610 standards run before and after each set of 16 samples) was applied to all sample acquisitions to correct for mass bias. The NIST 612 was used for ^137^Ba, ^135^Ba, ^87^Sr, ^86^Sr and ^26^Mg and NIST 610 for ^134^Ba and ^136^Ba. Isotope ratios are expressed as the enriched isotope divided by the most commonly abundant isotope for each element used so that the measure of enrichment is always expressed as an increase in the enriched isotope relative to the most common isotope. Statistical analyses were conducted on the final post-processed acquisition data values.

**Statistical analysis.** Mark success for each treatment was evaluated using a mark detection threshold set by Warren-Myers et al. [23]. Briefly, the mark detection threshold for the isotope ratios ^134^Ba:^138^Ba, ^135^Ba:^138^Ba, ^136^Ba:^138^Ba, ^137^Ba:^138^Ba, ^86^Sr:^88^Sr, ^87^Sr:^88^Sr and ^26^Mg:^24^Mg were calculated from the average isotope ratios of fish across the control treatment (i.e. non-enrichment treatment) (S1 Dataset). To ensure a correct classification probability of 99.94%, mark detection thresholds were set at 3.3 standard deviations above the mean observed ratio in control fish for each enriched isotope used. Because of the inherent instability in isotopic ratios measured on single-detector, ICP-based mass spectrometers, we conservatively set the criteria for detecting a successful mark in the otolith as at least three consecutive acquisitions with ratios above the detection threshold.

Mark strength of ^134^Ba:^138^Ba, ^135^Ba:^138^Ba, ^136^Ba:^138^Ba, ^137^Ba:^138^Ba, ^86^Sr:^88^Sr, ^87^Sr:^88^Sr and ^26^Mg:^24^Mg for each isotope enrichment concentration used (1, 0.1, 0.01, & 0.001 μg isotope g^-1^ fish) was analysed using a series of ANOVAs with isotope concentration and combination treated as fixed factors. The response variables used were the maximum isotope ratio observed (intensity) and the numbers of acquisitions above detection (spatial extent), in each fish otolith. The number of acquisitions above detection were ln (count + 1) transformed to improve ANOVA assumptions of equal variances.

The effects of treatment on fish length (fork length), weight and condition over the experimental period were analysed with one-way ANOVAs. The response variables used were change in fish length, weight and condition over the time frame of the experiment (sampling at 90 days and harvest 570 days). Fish condition was estimated using Fulton’s condition factor (*K*) calculated with the formula K = ((W/L3)×100), where W is the live body weight (g), and L is the fork length (cm) [27].

## Results

### Mark success

**Fig 1:**
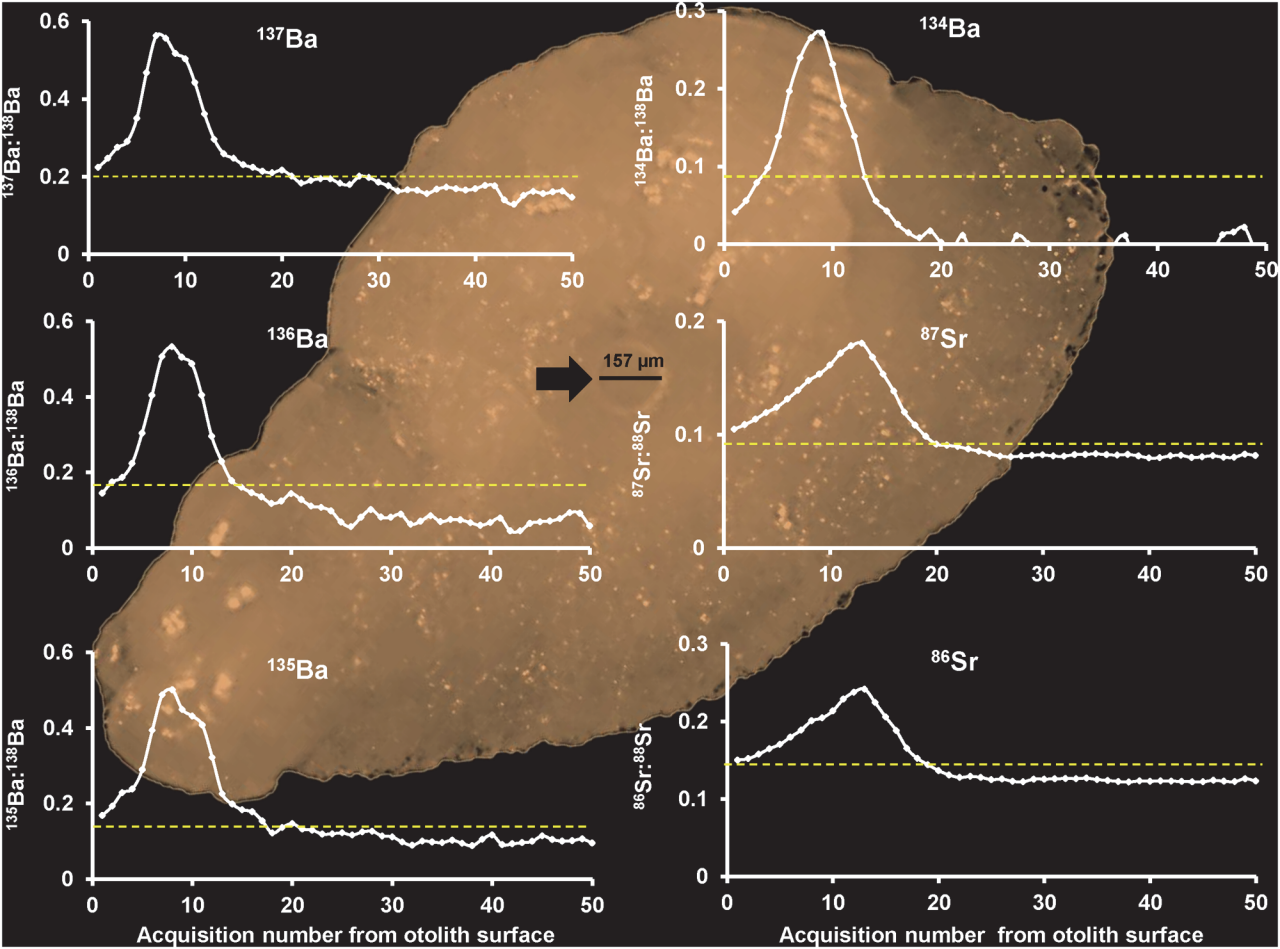
Six mark enriched stable isotope fingerprint. Scans of six enriched isotope markers, ^137^Ba, ^136^Ba, ^135^Ba, ^134^Ba, ^86^Sr and ^87^Sr in the otolith of Atlantic salmon parr successfully delivered during vaccination at a concentration of 1 μg. g^-1^ fish for Sr isotopes and 0.01 μg. g^-1^ fish for Ba isotopes. White lines represent the first 50 acquisition values recorded for each isotope analysed using LA-ICP spot ablation with a spot size diameter of 157 μm (depicted by black arrow). Ablation began from the surface of the otolith and ablated towards to core. Yellow dotted lines show the 99.94% mark detection threshold for determining a unique mark for each isotope used.

A six marker fingerprint combination using the enriched stable isotopes ^137^Ba, ^136^Ba, ^135^Ba, ^134^Ba, ^86^Sr and ^87^Sr was successfully created by marking during vaccination (Fig. 1). However, mark success was dependent on enrichment concentration and isotope combination (Table 2). ^137^Ba achieved 100% mark success with a minimum concentration of 0.001 μg. g^-1^ fish when used as a single isotope marker and 0.01 μg. g^-1^ fish when used in a combination of 4 or 7 isotopes. Marking with ^135^Ba and ^136^Ba was 100% successful with a minimum concentration of 0.01 μg. g^-1^ fish when used in combinations of 4 or 7 isotopes. Marking with ^134^Ba was 100% successful with a minimum concentration of 0.01 μg. g^-1^ fish when used in a 7 isotope combination. Sr isotopes were only successful at a concentration of 1 μg. g^-1^ fish. Mark success using ^86^Sr was 100% successful in combinations of 4 or 7 isotopes, and ^87^Sr was 100% successful in the 7 isotope combination. ^26^Mg used in a combination of 7 isotopes produced no successful marks at any concentration level.

**Table 2:** Mark success during vaccination using multiple combinations of isotope markers.

	Conc.	Isotope mark success						
Combination	(μg. g^-1^ fish)	^26^Mg	^86^Sr	^87^Sr	^134^Ba	^135^Ba	^136^Ba	^137^Ba
7	1	0%	**100%**	**100%**	**100%**	**100%**	**100%**	**100%**
	0.1	0%	22%	56%	**100%**	**100%**	**100%**	**100%**
	0.01	0%	0%	0%	**100%**	**100%**	**100%**	**100%**
	0.001	0%	0%	0%	0%	70%	20%	70%
4	1	-	**100%**	-	-	**100%**	**100%**	**100%**
	0.1	-	30%	-	-	**100%**	**100%**	**100%**
	0.01	-	0%	-	-	**100%**	**100%**	**100%**
	0.001	-	0%	-	-	80%	20%	80%
1	1	-	-	-	-	-	-	**100%**
	0.1	-	-	-	-	-	-	**100%**
	0.01	-	-	-	-	-	-	**100%**
	0.001	-	-	-	-	-	-	**100%**

Mark success during vaccination using combinations of 1, 4 or 7 isotopes at one of four concentrations (1, 0.1, 0.01 & 0.001 μg per g fish weight). Mark success was classed as three consecutive isotope ratios 3.3 standard deviations above control ratios for each isotope used.

### Intensity of isotope enrichment

**Fig 2:**
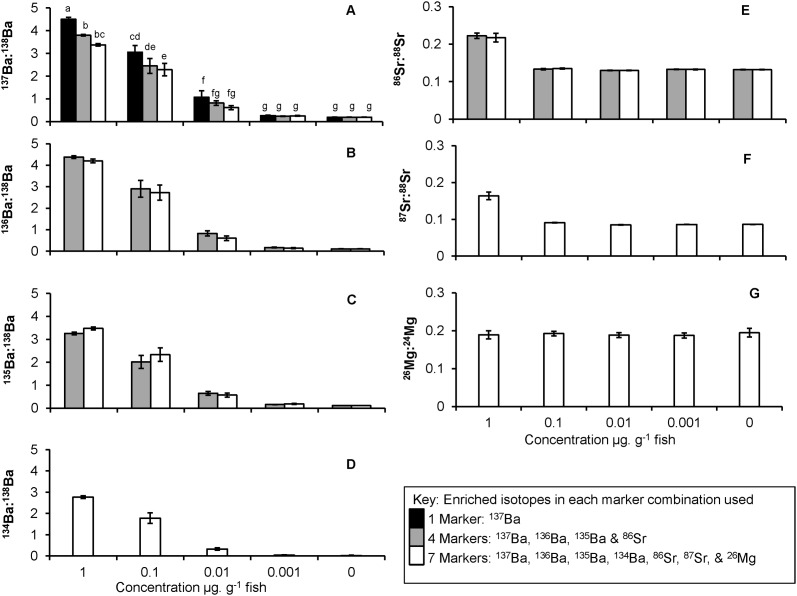
Intensity of mark using multiple combinations of isotopes. Maximum isotope ratios for ^137^Ba (A), ^136^Ba (B), ^135^Ba (C), ^134^Ba (D), ^86^Sr (E), ^87^Sr (F) and ^26^Mg (G) when used either singularly or in combination with 4 or 7 isotope markers. Bars represent mean maximum ratio for each concentration by isotope combination treatment. Error bars represent ± 1 SE. Concentrations were 1, 0.1, 0.01, 0.001 or 0 (control) μg isotope g^-1^ fish for each isotope used in a treatment. Letters above bars for ^137^Ba (A) show the Post Hoc Tukey HSD for the interaction term (Concentration*Combination, *p* < 0.05), different letters mean bars are significantly different.

^**137**^ **Ba,** ^**136**^ **Ba,** ^**135**^ **Ba and** ^**134**^ **Ba max isotope ratios.** For ^137^Ba, an interaction between marker combination and the concentration of isotope used, showed that as concentration decreased and marker combination increased, mark strength decreased (interaction term: concentration x combination, F_6, 127_ = 3.01, *p* = 0.009). Post hoc test for the interaction term highlighted that ^137^Ba used as a singular marker produced higher maximum ratios than combinations of 4 or 7 isotopes, depending on the enrichment concentration used (Fig. 2A, Tukey HSD: 1 μg, 1 marker > 4 markers = 7 markers; 0.1 μg, 1 marker > 7 markers; *p* < 0.05).

There was no interaction between combination and concentration for ^136^Ba or ^135^Ba (F_3, 87_ = 0.2 & 1.2 respectively, *p* > 0.3 for both). Mark strength for ^136^Ba and ^135^Ba used in combinations of 4 or 7 isotopes decreased as concentration decreased (Fig. 2B, F_3, 87_ = 341, *p* < 0.001 and Fig. 2C, F_3, 87_ = 337, *p* < 0.001 respectively; Tukey HSD: 1 μg > 0.1 μg > 0.01 μg > 0.001 μg = 0 μg, *p* < 0.05 for both). However, there was no difference in mark strength for either isotope when they were used in combinations of 4 or 7 (^136^Ba, F_1, 87_ = 2.11, *p* = 0.15; ^135^Ba, F_1, 87_ = 2.24, *p* = 0.14). ^134^Ba produced a similar pattern as observed with the other Ba isotopes of decreased mark strength as concentration decreased when used in a 7 marker combination (Fig. 2D, F_4, 48_ = 178, *p* < 0.001; Tukey HSD: 1 μg > 0.1 μg > 0.01 μg > 0.001 μg = 0 μg, *p* < 0.05).

^**86**^ **Sr and** ^**87**^ **Sr max isotope ratios.** There was no interaction between combination and concentration for ^86^Sr (F_3, 87_ = 0.2, *p* = 0.8). Mark strength for ^86^Sr was 1.6 times stronger in the highest concentration (1 μg. g^-1^ fish) compared to the 3 lower concentrations and the control (Fig. 2E, F_3, 87_ = 229, *p* < 0.001; Tukey HSD: 1 μg > 0.1 μg = 0.01 μg = 0.001 μg = 0 μg, *p* < 0.05) and there was no difference in mark strength between the 4 or 7 isotope combinations (F_1, 87_ = 0.098, *p* = 0.76). Similarly, ^87^Sr used in a 7 marker combination produced 1.9 times stronger marks in the highest concentration (1 μg. g^-1^fish weight) compared to the 3 lower concentrations and the control (Fig. 2F, F_4, 48_ = 74, *p* < 0.001; Tukey HSD: 1 μg > 0.1 μg = 0.01 μg = 0.001 μg = 0 μg, *p* < 0.05).

^**26**^ **Mg max isotope ratios.** No difference in mark strength across concentrations was observed for ^26^Mg when used in the 7 marker combination enrichment (Fig. 2G, F_4, 48_ = 0.17, *p* = 0.96).

### Spatial extent of isotope enrichment

Analysis of the total number of acquisitions observed above the detection threshold were only analysed for the Ba and Sr enrichment treatments. Mg enrichment did not produce enough acquisitions above the detection threshold to warrant further analyses. No control fish had 3 consecutive acquisitions above the detection threshold for any Ba or Sr isotope, so the analysis was restricted to the isotope enriched vaccine treatments.

**Fig 3:**
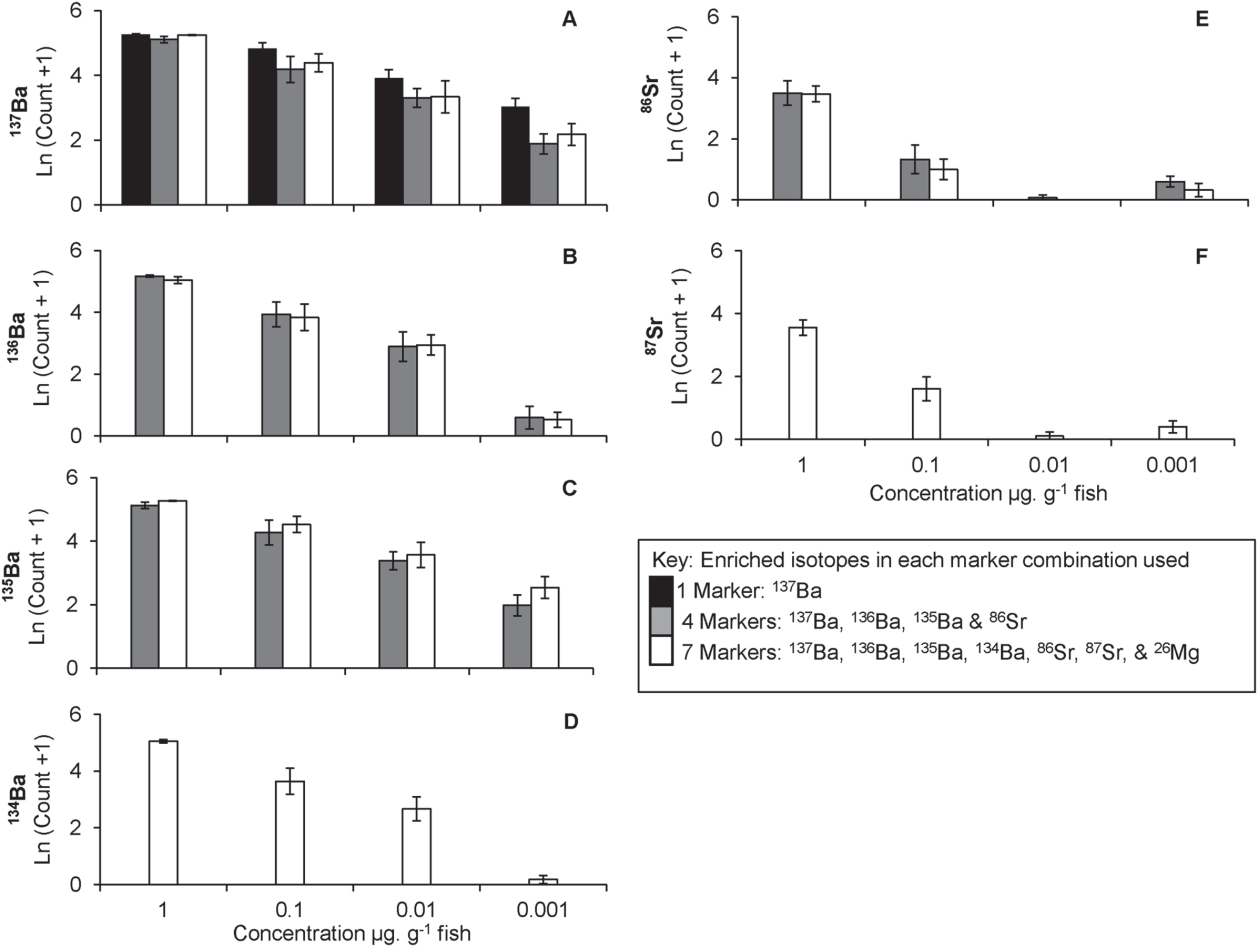
Spatial extent of mark using multiple combinations of isotopes. Number of acquisitions above detection for ^137^Ba (A), ^136^Ba (B), ^135^Ba (C), ^134^Ba (D), ^87^Sr (E) and ^86^Sr (F) when used singularly, or in a combination with 4 or 7 isotope markers. Bars represent mean Ln (count +1) values for each concentration by isotope combination treatment. Error bars represent ±1 SE. Note: there is no 0 concentration treatment as no control fish had 3 consecutive acquisitions above the detection threshold.

^**86**^ **Sr and** ^**87**^ **Sr acquisitions above detection.** There was no interaction between combination and concentration for ^86^Sr (F_3, 77_ = 0.2, *p* = 0.8).The number of acquisitions above detection for ^86^Sr were 10 times higher in the high concentration (1μg. g^-1^ fish) compared to the 3 lower concentrations (Fig. 3E, F_3, 77_ = 86.6, *p* < 0.001; Tukey HSD: 1 μg > 0.1 μg = 0.01 μg = 0.001 μg, *p* < 0.05). No difference was detected in the number of acquisitions between the 4 or 7 isotope combinations (F_1, 77_ = 1.09, *p* = 0.3). Similarly, ^87^Sr used in a 7 marker combination produced 10 times more acquisitions in the 1μg. g^-1^fish concentration and 2.5 times higher in the 0.1μg. g^-1^fish concentration compared to the two lowest concentrations (0.01 and 0.001 μg) (Fig. 3F, F_3, 38_ = 56.8, *p* < 0.001; Tukey HSD: 1 μg > 0.1 μg > 0.01 μg = 0.001 μg, *p* < 0.05).

^**137**^ **Ba,** ^**136**^ **Ba,** ^**135**^ **Ba and** ^**134**^ **Ba acquisitions above detection.** There were no interactions between concentration and combination for ^137^Ba (F_6, 117_ = 0.85, *p* = 0.5), ^136^Ba or ^135^Ba (F_3, 87_ = 0.04 & 0.29 respectively, *p* > 0.5 for both). Separately, isotope concentration and combination did influenced the total number of acquisitions above the detection threshold for ^137^Ba (F_3, 117_ = 77.9, *p* < 0.001 and F_2, 117_ = 7.56, *p* = 0.001, respectively). Count ratios were highest for ^137^Ba when used as a single marker at the highest concentration (1 μg. g^-1^ fish), but decreased as enrichment concentration decreased (Fig. 3A; Tukey HSD: 1 μg > 0.1 μg > 0.01 μg > 0.001 μg, *p* < 0.05) and as marker combination increased from 1 to 4 or 7 isotopes (Tukey HSD: 1 marker > 4 markers = 7 markers, *p* < 0.05). Total number of acquisitions for ^136^Ba, ^135^Ba and ^134^Ba used in isotope combinations of 4 and 7, or only 7, were affected by concentration (F_3,77_ = 95.9, F_3,77_ = 51.1, F_3,38_ = 38.1, respectively; *p* < 0.001 for all). As concentration decreased, total count ratios decreased accordingly (Fig. 3B–3D; Tukey HSD: 1 μg > 0.1 μg > 0.01 μg > 0.001 μg, *p* < 0.05).

### Mortality and growth

**Fig 4:**
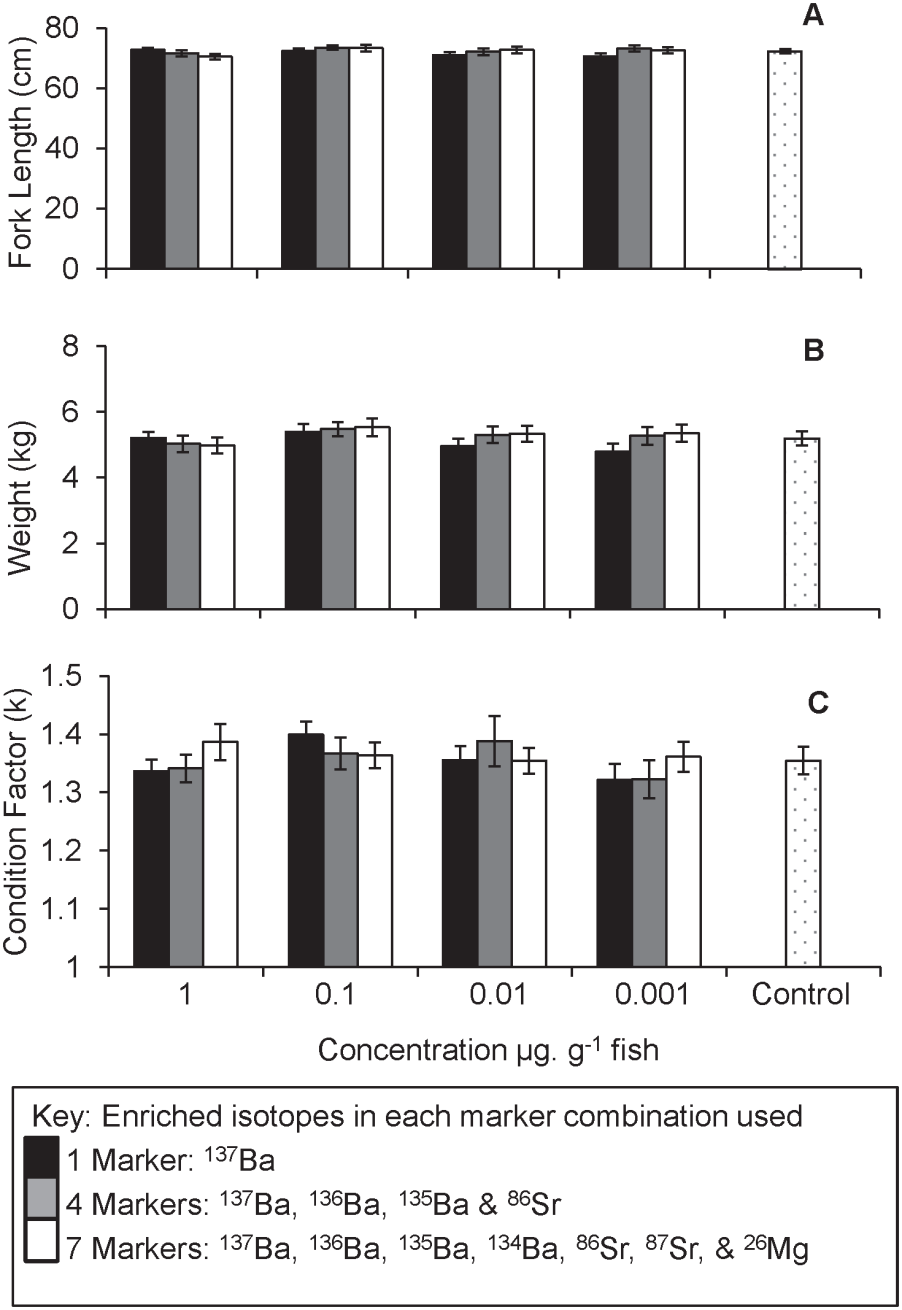
Comparison of growth parameters at harvest. Graphs show average fork length (A), weight (B), and Fulton’s condition factor K (C), for all fish at harvest in each of the treatments and the control. Error bars show ± 1 Standard Error.

There was no effect of treatment on mortality, with only 3 mortalities out of a total of 650 injected fish during the first 90 days before sea-transfer. Prior to sea transfer, overall, experimental fish increased in weight by 53.9 ± 0.6 g (mean ± SE) and fork-length by 3.9 ± 0.02 cm. No differences were detected among treatments for fish growth (weight: F_12, 646_ = 1.18, *p* = 0.3; fork length: F_12, 646_ = 1.27, *p* = 0.2) or fish condition (Fulton’s condition factor (*k*): F_12, 646_ = 1.02, *p* = 0.4). However, average condition of fish across all treatments was approximately 10% lower at 90 days post injection compared to day 1 (Fulton’s condition factor (*k*): Day 1 = 1.30 ± 0.002; Day 90 = 1.16 ± 0.003). Fish harvested at 570 days post injection (5.21 ± 0.06 kg, fork-length 72.2 ± 0.3 cm, condition factor (*k*) 1.36 ± 0.007) showed no difference in length, weight or condition among treatments (Fig. 4, weight: F_12, 408_ = 1.11, *p* = 0.3; fork length: F_12, 408_ = 0.84, *p* = 0.6; Fulton’s condition factor (*k*): F_12, 408_ = 0.82, *p* = 0.6); *p* = 0.4, 0.6, 0.6 respectively). Mortality per treatment during the sea cage stage averaged 7.4 ± 1%, with no difference among treatments (χ^2^_12_ = 7.2, *p* > 0.1).

## Discussion

### Mark success and strength

We have successfully produced a six marker stable isotope fingerprint and effectively determined the minimum optimal concentrations required for the 63 possible combinations of four Ba isotopes and two Sr isotopes for fingerprint marking Atlantic salmon otoliths during vaccination (Fig 1.). Creating a single Ba isotope fingerprint mark using ^137^Ba with 100% mark success is achievable at a concentration of 0.001 μg. g^-1^ fish. However, as the number of isotopes used in a fingerprint combination increases to four (^137^Ba, ^136^Ba, ^135^Ba & ^86^Sr) or seven (^137^Ba, ^136^Ba, ^135^Ba, ^134^Ba, ^87^Sr, ^86^Sr & ^26^Mg) the required concentration of each Ba isotope needed to ensure 100% mark success increases to 0.01 μg. g^-1^ fish. Sr isotopes used in multiple fingerprint marks required higher concentrations in comparison to Ba isotopes (1 μg vs 0.01 μg. g^-1^ fish, respectively) to guarantee 100% mark success, and for Sr there was no difference between fingerprint combinations of four or seven. These results demonstrate that the minimum concentration of isotope required to mark an individual fish during vaccination is 2 (for Sr) to 2000 (for ^137^Ba) times lower than the initial concentrations of 2 μg. g^-1^ fish trial by Warren-Myers et al. [23], depending on the isotope used and the isotope combination. This suggests that stable isotope marking during vaccination with Ba isotopes, in particular, has the potential to be economically feasible at an industry scale where costs per fish must be as low as possible.

Mark strength, measured using maximum isotope ratios (intensity) and number of acquisitions above detection (spatial extent) declined as isotope concentration was reduced. However, ratios did not decrease by an order of magnitude as one would predict, and for the Ba isotopes the intensity of marks was higher for single mark compared to 4 or 7 multiple marks at the highest concentration (1 μg. g^-1^ fish). This would suggest there is a possible facilitation, competition, or dilution effect influencing the degree of marker incorporation in multiple marker fingerprints above a threshold concentration. Facilitation of Ba uptake when Sr is present is known to occur in some fish species when the Sr:Ca ambient concentration in brackish or sea water is greater than 20 μmol. mol^-1^ [28]. However, if facilitation was occurring this should have increased the intensity of Ba marks when Sr was present in multiple mark fingerprints, not decreased mark intensity. Reduced mark intensity when using multi-isotopes markers compared to a single marker due to competition has not been reported before, but maybe a plausible explanation if 1 μg. g^-1^ fish (highest concentration tested) is a threshold at which competition among isotopes of the same element occurs. Dilution is most likely the cause for reduced mark intensity when multiple isotopes from the one element (e.g. ^137^Ba, ^136^Ba, ^135^Ba, ^134^Ba) are used together. A dilution effect could result from the residual amount of ^138^Ba impurities in the enrich isotopes used (^138^Ba impurities in: ^137^Ba = 17.4%; ^136^Ba = 2.4%; ^135^Ba = 3.6%; ^134^Ba = 5.3%, Oak Ridge National Laboratory; www.ornl.gov). A dilution effect of added residual ^138^Ba would also explain why mark success for ^137^Ba, which was 100% when used as a single isotope marker at the lowest concentration (0.001 μg. g^-1^ fish), dropped to 80% when used in combination with 2 other Ba isotope markers (^135^Ba, ^136^Ba) and to 70% when used with 3 other Ba isotope markers (^135^Ba, ^136^Ba, ^134^Ba). Hence, an increase in marker concentration from 0.001 to 0.01 μg. g^-1^ fish for multiple Ba marks is required to ensure mark intensity and spatial extent is strong enough that 100% unique marks are created.

Mark success with ^26^Mg was unsuccessful even at the highest concentration of 1 μg. g^-1^ fish. Poor mark success with Mg isotopes has been reported for marking via vaccination [23], or larval immersion [26] and may be due to self-regulation of Mg in fish [29] or a slow exchange rate of Mg ions into the endolymph fluid that surrounds the otolith [30]. Alternatively, natural levels of Mg in water and food may be too high for the introduction of an enriched Mg spike to significantly shift the natural Mg isotope ratios at the concentrations Mg has been tested. Greater concentrations than used in this study, even if successful, would make Mg too costly and hence unsuitable for marking farmed salmon.

### Fish condition and survival

Stable isotope marking with Ba, Sr and Mg did not affect growth, condition or survival, among treatments over the 570 days between injection date and harvest date. This is consistent with other stable isotope marking studies that used transgenerational and larval immersion techniques, and which similarly detected no negative effects on survival and growth due to marking [25,26,31,32]. Although average condition of all fish in the experiment dropped initially (~10%) over the first 90 days, the photoperiod regime used in the trial typically induces a decrease in condition factor similar to that normally seen during the parr–smolt transformation of Atlantic salmon and other salmonid species [33,34]. In addition, growth rates often reduce in the short-term in vaccinated Atlantic salmon [35,36], which is associated with loss of appetite post vaccination [37]. At harvest, fish condition was slightly higher compared to condition at injection date and no differences were found among treatments suggesting there are no long-term detrimental effects of stable isotopes on Atlantic salmon when delivered during vaccination.

### Application of otolith fingerprinting with enriched stable isotopes during vaccination

We have demonstrated that isotope marking delivered during vaccination can effectively mark farmed salmon and enable detection of the mark with 99.94% accuracy. Moreover, the concentrations required are sufficiently low that cost-effectiveness is high compared to all other common salmonoid mass marking techniques (Table 3). The amount of isotope required to mark a fish delivered during vaccination is between 0.01 and 0.001 μg. g^-1^ fish for Ba isotopes and 1 μg. g^-1^ fish weight for Sr isotopes. Typically, Atlantic salmon parr average 40 g at vaccination time, meaning the total amount of enriched isotope required for marking ranges between 0.04 and 1.6 μg of Ba isotope per fish depending on the fingerprint combination used (Table 4) and 40 μg per fish for a single Sr isotope. The optimal isotope concentration delivered during vaccination used in the present study is lower for Ba, but higher for Sr when compared with a larval immersion study on Murray cod (*Maccullochella peelii*) [25], which used the equivalent of 2 to 3 μg of Ba, and 5 to 8 μg of Sr per individual. Hence, for marking during vaccination at the concentrations we have demonstrated, isotopes of Ba are the most suitable and cost effective given current prices (isotope source = Oak Ridge National Laboratory; www.ornl.gov), which range from $US 0.0002 to $US 0.02 per fish depending on the combination of Ba isotopes used. Concentrations at which Sr isotopes are effective render them less economically viable for delivery during vaccination (from $US 0.48 to $US 1.72 per fish depending on the combination of Sr isotope). Sr isotopes may be more cost effective for marking using other techniques, such as immersion with osmotic induction (e.g. de Braux et al. [38]).

**Table 3:** Marker costs for mass marking Atlantic salmon.

Method	Marker cost ($US) per fish	Product information source
Coded wire tag	0.09	http://www.nmt.us
Elastomer tags	0.09	http://www.nmt.us
Pit tags	2.50	http://bts-id.com
Adipose fin clipping	0.05	http://wdfw.wa.gov
Ba isotope marking during vaccination	0.0002–0.02	http://www.ornl.gov

Marker cost per fish refers to the material cost of the marker or tag, except in the case of adipose fin clipping were the cost relates to the cost of removing the adipose fin per fish

**Table 4:** Ba isotope otolith fingerprinting.

		Required amount of isotope (μg per 40g fish)				Total	Cost ($US)
Code #	Isotope combination	^137^Ba	^136^Ba	^135^Ba	^134^Ba	per fish	per fish
1	^137^Ba	0.04	-	-	-	0.04	0.0002
2	^137^Ba, ^136^Ba	0.4	0.4	-	-	0.8	0.0055
3	^137^Ba, ^135^Ba	0.4	-	0.4	-	0.8	0.0086
4	^137^Ba, ^134^Ba	0.4	-	-	0.4	0.8	0.0122
5	^137^Ba, ^136^Ba, ^135^Ba	0.4	0.4	0.4	-	1.2	0.0119
6	^137^Ba, ^136^Ba, ^134^Ba	0.4	0.4	-	0.4	1.2	0.0155
7	^137^Ba, ^135^Ba,^134^Ba	0.4	-	0.4	0.4	1.2	0.0187
8	^137^Ba,^136^Ba,^135^Ba,^134^Ba	0.4	0.4	0.4	0.4	1.6	0.0219
9	^136^Ba	-	0.04	-	-	0.04	0.0003
10	^136^Ba,^135^Ba	-	0.4	0.4	-	0.8	0.0098
11	^136^Ba, ^134^Ba	-	0.4	-	0.4	0.8	0.0133
12	^136^Ba, ^135^Ba,^134^Ba	-	0.4	0.4	0.4	1.2	0.0198
13	^135^Ba	-	-	0.04	-	0.04	0.0006
14	^135^Ba,^134^Ba	-	-	0.4	0.4	0.8	0.0165
15	^134^Ba	-	-	-	0.04	0.04	0.0010

Minimum required amounts (μg) and estimated raw material cost per fish ($US, Oak Ridge National Laboratory; www.ornl.gov) for stable isotope marking of 40 g Atlantic salmon parr during vaccination.

**Country production and data source:** **Scotland.** Annual production 180 000 tonnes, 13 companies, *Scottish Salmon Producers’ Organisation Limited (SSPO)* Website; www.scottishsalmon.co.uk. **Canada** Annual production 100000 tonnes, 6 companies, *Canadian Aquaculture Industry Alliance (CAIA)* Website; www.aquaculture.ca **Faroe Islands.** Annual production 61000 tonnes, 3 companies, *Faroe Fish Farmers Association (FFFA)* Website; www.salmon.for **Norway.** Annual production 1.28 Million tonnes, 83 companies, *The Norwegian Ministry of Fisheries and Coastal Affairs* (*NMFCA*) Website; www.fisheries.no

In addition to marking, there is an additional analysis cost of identifying marked fish. The cost per sample to analyse based on this study this was between $US 15 to 20 per fish. Hence, analytical costs for monitoring for compliance of correct application of marks at an individual fish farm, or an assessment of a group of fish thought to have come from an escape event could be done for as little as $US 300 to 400 (N = 20 fish) due to the high accuracy (99.94%) of the enriched stable isotope marking method.

### Industry-scale marking with isotopes of Ba

For a mass marking technique to work at an industry scale and to be successful in driving compliance, escaped marked fish need to be traceable to a point that assigns accountability for an escape event. Stable isotope fingerprint marking could ensure accountability if each company within major producer nations was assigned its own unique marker combination. Mass marking would also allow for an accurate assessment of the level of integration between escapees and wild fish. For example, the Scottish salmon industry produces 180 000 tonnes of salmon a year from 13 main companies, hence, using the 13 cheapest of a possible 15 Ba marker combinations (Table 4) would enable each company in Scotland to have its own unique salmon identification mark at a median cost of $US 0.012 per fish. Canadian salmon farming produces 100 000 tonnes of salmon per year from 6 main companies, meaning only the 6 cheapest of a possible 15 Ba marker combinations are required to mark at the company level for the Canadian salmon industry at a median cost of $US 0.0008 per fish. The Faroe Islands produces 61 000 tonnes of salmon per year from just 3 companies, requiring just 3 unique Ba markers at a median cost of $US 0.0003 per fish.

Currently the biggest producer of salmon worldwide is Norway, with an estimated annual production of 1.28 million tonnes per year from 83 companies. 15 Ba markers are insufficient to assign a unique mark at the company level. More markers would need to be tested, for example ^132^Ba and ^131^Ba to ensure enough unique combinations. However, recent legalisation in Norway now allows for greater industry amalgamation; individual stakeholders may now obtain up to a 40% share of Norway’s total production (increased from 25%). Hence, if the total number of companies is reduced in the future to less than 60 through the amalgamation of smaller industry partners, marking with Ba and Sr stable isotopes during vaccination would become viable for the Norwegian salmon industry. An alternate solution that could produce hundreds of marks using only the most cost-effective barium isotopes would be to combine marking during vaccination at the parr stage (this study) with marking via immersion during the larval stage [38] to produce multiple fingerprint marks in different parts of the otolith. Although confirmation that this approach doesn’t cause cross-contamination of marks is required, it would allow for a possible 255 unique fingerprints.

We have shown that stable isotope fingerprint marking during vaccination using isotopes of Ba is an economically viable method for uniquely identifying fish to the company level for the major salmon production regions worldwide. Importantly, the marks are permanent, unique, relatively easy to detect, and can be incorporated into standard salmon hatchery production with no additional production or welfare issues for fish grown to full commercial production size.

## Supporting Information

S1 Dataset Data summary. (XLSX)
